# Effect of Electrode Tuning on the Resistive Switching Behaviour of MXene-Based Composites

**DOI:** 10.3390/polym17101309

**Published:** 2025-05-11

**Authors:** Maria Grácio, Henrique Teixeira, Catarina Dias, João Ventura

**Affiliations:** IFIMUP, Department of Physics and Astronomy, Faculty of Sciences, University of Porto, Rua do Campo Alegre, s/n, 4169-007 Porto, Portugal; maria.gracio@fc.up.pt (M.G.); hteixeira@fc.up.pt (H.T.); cdias@fc.up.pt (C.D.)

**Keywords:** 2D materials, conductive filaments, MXene, polymeric composites, resistive switching

## Abstract

As new materials are required to overcome the challenges presented by non-volatile resistive switching (RS) devices, organic based composites are gaining attention due to their easy preparation, tunability, scalability and good mechanical properties. Here, we study how the interaction between MXene and Polyvinylidene fluoride (PVDF) contribute to the RS phenomenon, and how different electrode materials [Ag (top) and W, ITO (bottom)] affect the conduction in these devices. Our detailed structural and electrical analyses of the composite layer reveals that the RS mechanism is related with the formation and rupture of conductive filaments pinned by the MXene sites. The W-based structure exhibits lower switching voltage (<1 V) and a higher ON/OFF resistance ratio of 15. In contrast, the ITO-based structure requires a higher bias voltage and displays a more gradual switching (ON/OFF ratio of 3), likely due to the competition between the migration of Ag^+^ ions and oxygen vacancies from ITO. This work paves the way in understanding how to exploit the integration of novel two-dimensional (2D) materials with polymers for neuromorphic computing.

## 1. Introduction

The swift development of electronics and data-storage technologies is accompanied by a growing need for new materials to overcome the challenges these research areas are currently facing, such as reproducibility, stability and miniaturization. In particular, resistive switching (RS) memories, based on metal-insulator-metal (MIM) structures, are an emerging candidate for new data storage and neuromorphic devices, due to their scalability, low-power operation and simple structure [1,2,3]. RS memories are non-volatile devices [4,5] that reversibly switch between low (ON) and high (OFF) resistance states (LRS/HRS), through SET (OFF to ON) and RESET (ON to OFF) processes. Nevertheless, the switching behaviour relies strongly on the properties of the components of the MIM structure, so that the study of new materials is crucial to enhance RS devices. Among the most successfully implemented in RS technology are metal oxides [6,7,8], perovskites [9,10,11] and 2D materials [12,13,14,15,16].

In particular, 2D materials present a unique layered structure that results in singular electrical, optical, thermal and mechanical properties [17,18]. Among the vast variety of 2D structures, MXenes are an emerging family of 2D carbide layered materials that possess unique electronic and optical properties for micro- or nanodevices, and have successfully been integrated in RS devices, demonstrating improved performance, superior neuromorphic characteristics and reduced power consumption [19,20,21,22]. MXenes are obtained by etching the A layer from the MAX phase (M_*n*+1_AX_*n*_), where M is an early transition metal, A is an A-group element, X is either carbon and/or nitrogen and *n* = 1–3 [23]. The general formula for MXenes is M_*n*+1_X_*n*_T_*X*_, making them transition metal carbides, nitrides, or carbonitrides, and in which $T_{X}$ corresponds to surface terminations, such as −OH, −O or −F. These materials present great chemical stability, large aspect ratio and excellent electrical conductivity [24,25,26]. Furthermore, they present good optical transport due to high Fermi level density of states [27] and adjustable surface terminations, enabling the tuning of their physical and chemical properties [24,28,29]. In spite of their large potential, challenges still lie ahead for a wider and more systematic implementation of MXenes in electronic devices. These include the high conductivity of MXenes (that may inhibit satisfactory properties by hindering retention and endurance performance), or their ambient stability. In fact, the long-term stability of devices is recurrently compromised due to the oxidation of the MXene sheets, which affects performance and leads to unpredictable behaviour over time, creating device variability. A popular approach to overcome this issue includes the integration of MXenes in heterostructures [30] or polymer materials [31].

Particularly, the integration of PVDF with MXenes has been extensively studied for application in electromagnetic shielding and energy harvesting [32,33,34]. Nevertheless, even though neuromorphic properties of such composites have been addressed [35], their application in such electronic devices has not yet been thoroughly explored. PVDF presents compelling properties to be applied in resistive switching memories, such as a high dielectric constant and low dielectric losses. Furthermore, it is a robust polymer under harsh conditions, presenting good chemical stability [36]. It is also mechanically and thermally stable, as well as non-toxic and bio-compatible [37], making it a prominent material.

In this work, we performed a study on the effect of different configuration of active and inert electrodes [Ag (top), ITO and W (bottom)] on the resistive switching properties (conduction mechanism and performance) of an organic composite based on MXene 2D materials. By integrating Ti_3_C_2_T_*x*_ on the PVDF polymer, we were able to show bipolar RS and non-volatile characteristics. A detailed structural analysis showed improved MXene stability over time, with no clear evidence of oxidation. We identify silver ions conductive filament formation/rupture as the main switching mechanism in these structures, directed by the MXene sites serving as nucleation points. Furthermore, we found that the bottom electrode plays a relevant part in this behaviour, by influencing the vacancies that dominate the filamentary behaviour.

## 2. Materials and Methods

### 2.1. Materials

MAX phase (Ti_3_AlC_2_; purity ≥ 99%) powder was purchased from Nanochemazone (Leduc, AB, Canada). Lithium fluoride powder (LiF; <100 μm, purity ≥ 99.98%), Polyvinylidene fluoride (PVDF; M_W_ ∼ 530,000) and PET/ITO flexible substrate were acquired from Sigma Aldrich (St. Louis, MO, USA). Dimethylformamide (DMF; purity ≥ 99.5%) and Hydrochloric acid (HCl; purity ≥ 37%) were acquired from Fisher Scientific (Hampton, NH, USA).

### 2.2. Synthesis of Ti_3_C_2_T_*x*_ MXene Flakes

The Ti_3_C_2_T_*x*_ MXenes used in this work were fabricated through the minimally intensive layer delamination (MILD) etching technique. HF is formed in-situ, by mixing 14.8 mL of HCl with 1.6 g of LiF powder. After 30 min of homogenization, 1 g of MAX powder is slowly added to the solution. The etching process was kept for 30 h, at 35 °C. Washing cycles (3500 rpm for 5 min in centrifuge) are performed, after exfoliation, to stop the etching process and remove byproducts, until neutral pH is obtained. Afterwards, MXenes were re-dispersed in ultra pure water, and subjected to 1 h sonication, to obtain delaminated flakes.

### 2.3. Fabrication MXene/PVDF Nanocomposite Structures

PVDF was dissolved in DMF with concentration of 10 wt% for 1 h at 60 °C, under magnetic stirring. For the Ti_3_C_2_T_*x*_:PVDF composite, after complete dissolution of the PVDF polymer, Ti_3_C_2_T_*x*_ flakes were added to the solution, with a 3 wt% Ti_3_C_2_T_*x*_ flakes in the polymer. The solution was stirred for another 30 min, for homogenization of the MXene flakes on the solution. Following this step, the composite was deposited on PET/ITO (150 nm) or Si/SiO_2_/W (50 nm) substrates by spin-coating. This deposition consisted of a 15 s step at 600 rpm for the spreading of the solution, plus a 45 s step at 4000 rpm for the ultimate definition of the thin film thickness. The evaporation of the solvent proceeded in an oven at 60 °C for 5 h. This way, thin films were produced with a thickness of around 0.8 μm, measured by Scanning Electron Microscopy (SEM) cross section. Finally, circular electrodes (Ag) of 300 μm in diameter and 50 nm in thickness, were deposited by ion beam deposition using a shadow mask.

### 2.4. Characterization

The crystalline structure of the thin film membranes was studied through X-ray Diffraction (XRD, Rigaku SmarLab (Tokyo, Japan)), using the SmartLab Rigaku diffractometer operated at 9 kW power (40 kV and 200 mA), with Cu Kα radiation (1.5406 Å) and the Bragg–Brentano θ/2θ configuration in the 2θ range of 5° to 60°, with a step of 0.01° and scan rate of 8°/s. Furthermore, Fourier-transform Infrared Spectroscopy Attenuated Total Reflectance (FTIR-ATR) using a Perkin Elmer Spectrum BX (PerkinElmer, Waltham, MA, USA) system, in transmission mode, with frequencies from 400 to 4000 ${\mathrm{cm}}^{-1}$, was used to study the molecular bonds in the polymer and MXenes. To analyze the surface morphology and cross-section of the deposited membranes, Scanning Electron Microscopy was performed with a High Resolution (Schottky) Environmental Scanning Electron Microscope with XRD Microanalysis (Quanta 400 FEG ESEM) and Electron Backscattered Diffraction analysis (EDAX Genesis X4M) (FEI, Hillsborough, OR, USA). The thickness of the samples was confirmed using cross-sectional images. Raman Spectroscopy was performed using an inVia Qontor Renishaw spectrometer (Renishaw, Wotton-under-Edge, England), with red (λ = 633 nm) and infrared (λ = 785 nm) lasers. The electrical characterization measurements were carried out at room temperature with tungsten microtips, using a sourcemeter (Keithley 2400C (Keithley Instruments, Cleveland, OH, USA)), with the bottom electrode grounded. Current compliance (I_*CC*_) was used to limit the maximum current.

## 3. Results and Discussion

### 3.1. Structure Characterization

**Figure 1:**
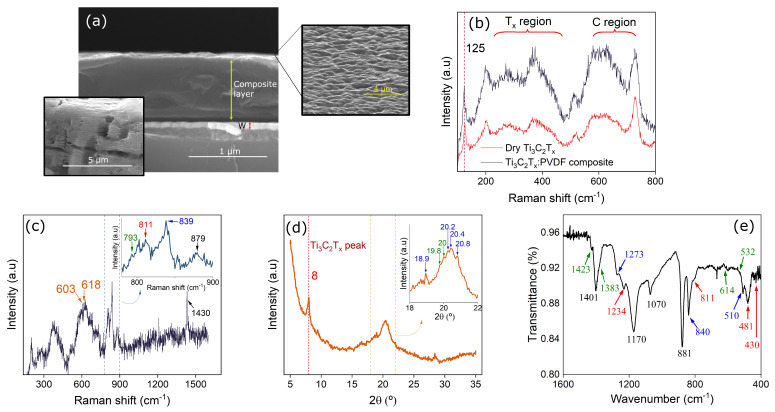
Structural characterization of the Ti_3_C_2_T_*x*_:PVDF active layer. (**a**) SEM cross section image of the layer, revealing a thickness of 0.8 μm. The inset on the left shows a SEM image of the MXene flakes. The inset on the right shows the surface of the composite after deposition. (**b**) Comparison of the Raman spectra of dry MXene flakes and composite layer measured using the 785 nm laser. (**c**) Raman spectra of composite layer measured using the 633 nm laser. (**d**) XRD and (**e**) FTIR spectra of the composite layer.

Figure 1a shows a cross section SEM image of a W/Ti_3_C_2_T_*x*_:PVDF/Ag structure, cut by freeze-drying. A thickness of approximately 0.8 μm is visible, together with a rough surface, supported by the inset on the right, that reveals the surface of the composite. The inset on the left shows a SEM image of Ti_3_C_2_T_*x*_ flakes, dried in a metallic surface, that clearly reveals the flakes multi-layer structure.

Figure 1b shows the spectra of dry MXene flakes measured 1 day after fabrication (red curve) with the 785 nm laser. Marked by curly brackets are the regions between 230–470 ${\mathrm{cm}}^{-1}$, assigned to the in-plane (E_*g*_) vibrations of the T_*x*_ layers, and 580–730 ${\mathrm{cm}}^{-1}$, assigned to both E_*g*_ and $E_{1g}$ carbon vibrations. MXenes tend to oxidize quite easily in contact with air, which represents a major disadvantage, since their electrical properties vary with the oxidation level. Therefore, we also studied the oxidation of the fabricated Ti_3_C_2_T_*x*_ MXene flakes embedded in the PVDF matrix, by measuring the spectra of the MXene composite 10 months after fabrication and exposure to ambient temperature and humidity conditions, also represented in Figure 1b (blue curve). It is clear that all the characteristic bands remain visible in the composite, and the 125 ${\mathrm{cm}}^{-1}$ resonance peak of MXenes is also observed. These results confirm the successful incorporation of MXenes in the PVDF polymer. Using the 633 nm laser to measure the composite, the MXenes naturally present a different characteristic spectra [Figure 1c], which can be useful to reveal their level of oxidation. The spectra shows the delaminated MXene [38] peak at 603 ${\mathrm{nm}}^{-1}$, and no formation of TiO_2_, since this would be revealed by the appearance of a peak near 154 ${\mathrm{cm}}^{-1}$, associated to the E_*g*_ mode of the TiO_2_ anatase phase. Only a slight red shift in the 620 ${\mathrm{nm}}^{-1}$ peak associated with oxidation [38] is observed. Furthermore, if considerable oxidation had occurred, the spectral region between 1000 and 1600 ${\mathrm{cm}}^{-1}$ should also become sizable compared to the other regions, which is not the case. We can thus conclude that, embedded in the PVDF matrix, there is no relevant oxidation of the MXene flakes after 10 months of fabrication. PVDF related bands are also visible [Figure 1c], being the 700–1400 ${\mathrm{cm}}^{-1}$ region the most relevant. In particular, the peak 793 ${\mathrm{cm}}^{-1}$ corresponds to the α phase, the peak at 839 ${\mathrm{cm}}^{-1}$ corresponds to the PVDF β phase, and the 811 ${\mathrm{cm}}^{-1}$ peak is associated to the γ phase. Peaks 879 and 1430 ${\mathrm{cm}}^{-1}$ are associated to the existence of the three phases.

Figure 1d shows the X-ray diffractogram of the composite thin film. The peak at 8° corresponds to Ti_3_C_2_T_*x*_, indicating that the MXenes are delaminated. The remaining sharp peaks correspond to the crystalline phases of PVDF, while the broader bands are an indication of its amorphous phase. We see the existence of at least two phases, being α at 2θ = 19.8, 20.0°; β at 2θ = 18.9, 20.2, 20.4, 20.8°. γ-phase peaks are not really visible, probably due to being hidden by close α and β peaks.

Figure 1e shows the FTIR measured absorption bands of the Ti_3_C_2_T_*x*_:PVDF film, as the crystalline phases of the PVDF can be further characterized by the infrared absorption bands comprised between 1500 and 400 ${\mathrm{cm}}^{-1}$. The peaks at 881, 1070, 1170 and 1401 ${\mathrm{cm}}^{-1}$ that commonly appear in all three phases are visible [39]. Peaks that are usually exclusively attributed to each of the three phases are also visible, being 532, 614, 1383, and 1424 ${\mathrm{cm}}^{-1}$ attributable to the α phase; 510, 840 and 1273 ${\mathrm{cm}}^{-1}$ to the β phase; and 430, 481, 811 and 1234 ${\mathrm{cm}}^{-1}$ to the γ phase. So, the three common phases of PVDF are present in the thin film. For the MXenes, there is no clear peak associated to their presence, as the main characteristic peaks coincide with the range of the PVDF major bands.

### 3.2. Electrical Characterization

**Figure 2:**
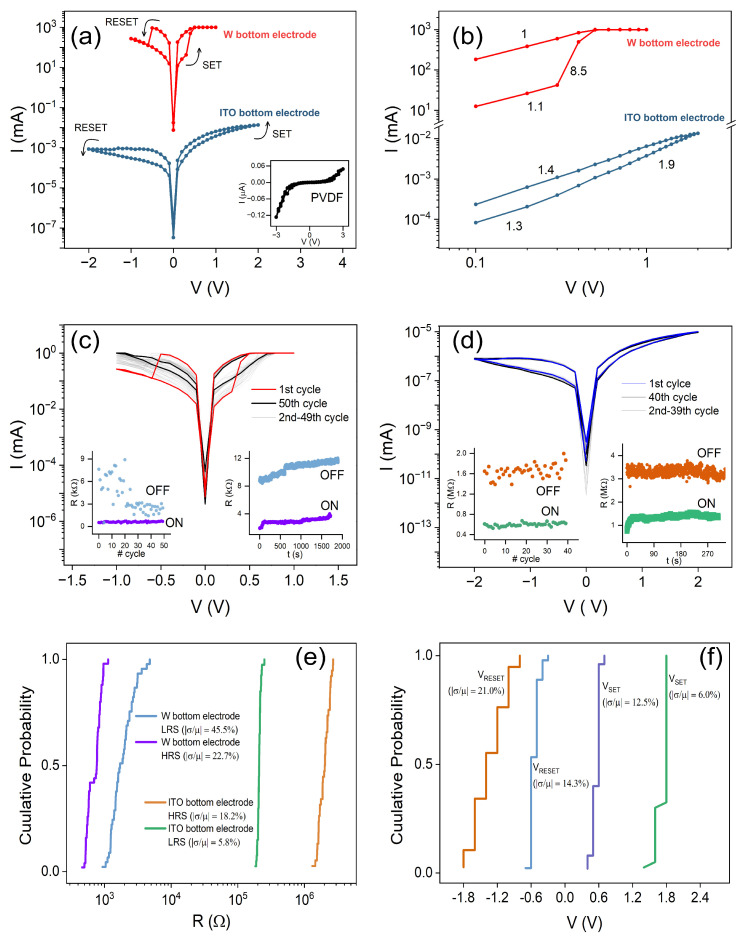
(**a**) I−V characteristics of W/Ti_3_C_2_T_*x*_:PVDF/Ag (red) and ITO/Ti_3_C_2_T_*x*_:PVDF/Ag (blue). The inset shows the I−V characteristic curve obtained for a W/PVDF/Ag control structure. (**b**) ln(I)−ln(V) characteristics of W/Ti_3_C_2_T_*x*_:PVDF/Ag (red) and ITO/Ti_3_C_2_T_*x*_:PVDF/Ag (blue). Cycling, endurance (left inset) and retention performance (right inset) of (**c**) W/Ti_3_C_2_T_*x*_:PVDF/Ag and (**d**) ITO/Ti_3_C_2_T_*x*_:PVDF/Ag. Cumulative probability of (**e**) resistance and (**f**) voltage in HRS and LRS, for both structures.

To study the effects of MXenes and electrode material on resistive switching, we fabricated W/Ti_3_C_2_T_*x*_:PVDF/Ag and ITO/Ti_3_C_2_T_*x*_:PVDF/Ag structures. The measured experimental I-V curves are represented in Figure 2a, revealing bipolar resistive switching for both W and ITO bottom electrodes. It is interesting to note that, from what has been observed, no electroforming process is required to obtain switching behaviour. In the W/Ti_3_C_2_T_*x*_:PVDF/Ag structure (red curve), the initial resistance is about 8 kΩ, and switching from low to high conductance (SET process) occurs at a bias of ≈0.3 V, reaching the current compliance of 1 mA, with a minimum resistance of 540 Ω, giving a ON/OFF ratio of 15. By sweeping the voltage to negative values, the high resistance state is recovered (RESET process) at −0.5 V. The characteristic RS curve of the ITO/Ti_3_C_2_T_*x*_:PVDF/Ag structure (blue curve) exhibits a much more gradual HRS to LRS transition, with no well defined $V_{SET}$ or $V_{RESET}$. The initial resistance is 1.2 MΩ, with SET process at positive bias leading to a resistance of 430 kΩ, giving an ON/OFF ratio of 3. Furthermore, the hysteresis is less pronounced, appearing more collapsed. The HRS is obtained by sweeping to negative voltage values (RESET process), also more gradually. As a control, we fabricated W/PVDF/Ag, with no MXenes. The inset of Figure 2a shows the corresponding I−V behaviour, clearly demonstrating the absence of hysteresis in the curve, with a initial resistance of approximately 500 MΩ. This indicates the critical role of the MXenes for the observation of resistive switching.

To explore the conduction mechanisms present in these structures, the I−V curves are represented in logarithmic scale in Figure 2b. For the W-based structure, the slope of the curve at low voltage is 1.1, showing a close to linear relation (I ∝ V) similar to the ohmic conduction model. As voltage increases, a sudden slope enhancement to 8.5 is observed near the SET voltage, indicating an abrupt increase in current. The structure then turns into the LRS, meaning that all the traps in the active layer are filled and the movement of free charges is enabled, increasing the current. By this stage, the curve has a slope of 1, indicating ohmic conduction. Concerning the ITO-based structure, the low voltage region between 0 and 0.3 V presents a slope of 1.3. In the 0.3 to 2 V range, the slope increases to 1.9. This is in agreement with the Child conduction mechanism (I ∝ $V^{2}$), during which the injection of more carriers is limited by the space charge of the active layer. As the voltage is swept back to zero, the slope is 1.4. By these analyses, we can attribute the conduction of the structure in the HRS to trap-filled space charge limited conduction (SCLC). Comparing the two curves in Figure 2b, it is clear that the ITO-based structure shows a much more gradual switching to the LRS.

Figure 2c,d show the cyclability, endurance, and HRS/LRS retention of the W and ITO-based structures, respectively. The W/Ti_3_C_2_T_*x*_:PVDF/Ag structure shows an endurance of over 50 cycles and a retention larger than 30 min. Concerning cycling, we see that the OFF resistance tends to gradually decrease after multiple cycles. The ITO/Ti_3_C_2_T_*x*_:PVDF/Ag structure shows an endurance of at least 40 cycles, with constant ON and OFF resistances. Concerning retention, the ON state has been kept for at least 5 min. The cumulative probability distribution confirms the small variability of the LRS for both structures [Figure 2e], and lower in general for the ITO bottom electrode structure. Concerning the cumulative probability for SET and RESET voltages [Figure 2f], it shows basically the same variability for SET and RESET with the W bottom electrode, and minimum for SET with ITO, while maximum for RESET.

**Figure 3:**
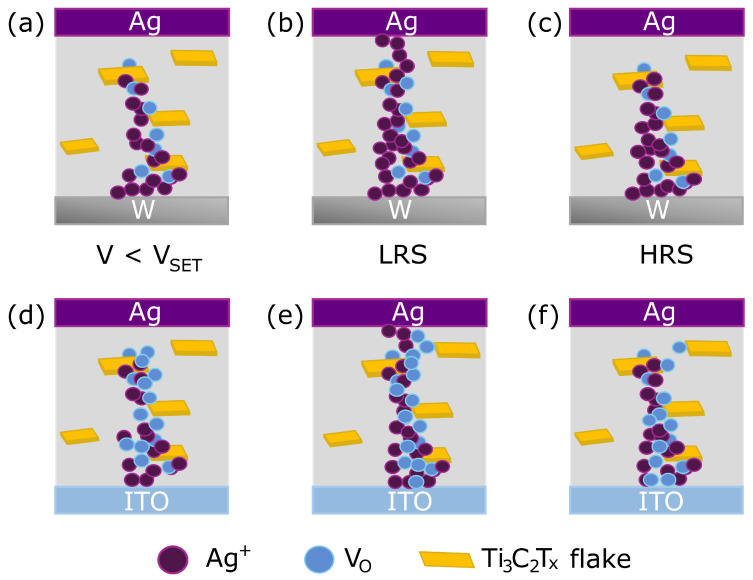
Schematic representation of the conduction mechanism interplay between Ag ions and oxygen vacancies present as MXene surface terminations in the (**a**–**c**) W/Ti_3_C_2_T_*x*_:PVDF/Ag and (**d**–**f**) ITO/Ti_3_C_2_T_*x*_:PVDF/Ag structures.

The resistive switching behaviours we obtained are influenced by both top active electrode and the presence of oxygen vacancy terminations in the MXene flakes, which also serve as nucleation centres, creating an interplay between the Ag^+^ ions and oxygen vacancies, as represented in Figure 3a–f, for W/Ti_3_C_2_T_*x*_:PVDF/Ag and ITO/Ti_3_C_2_T_*x*_:PVDF/Ag, respectively. This is the main difference from the single PVDF layer that did not show resistive switching. Concerning the top electrode effect, as voltage is increased, Ag atoms are oxidized, forming Ag^+^ ions that migrate towards the bottom electrode, eventually forming a filament that allows the free flow of charges in the LRS, leading to ohmic conduction. The MXene flakes serve as trapping sites in the dielectric layer that allow the nucleation of ions. Furthermore, the application of an electric field also creates oxygen vacancy movement that will drift and try to form a filament of vacancies, creating two competing mechanisms.

In fact, as seen above, the ITO-based structure displays a softer transition into LRS, indicating the relevance of oxygen vacancies role, in competition with the Ag filament formation. This is probably due to the ITO layer serving as an oxygen reservoir that feeds this competing mechanism and leads to a less pronounced switching.

## 4. Conclusions

In summary, conductive filament-based resistive switching is demonstrated in Ti_3_C_2_T_*x*_:PVDF composites, revealing the crucial role of MXenes to enable this effect. Furthermore, PVDF improves the stability of the MXene flakes, which show no oxidation up to 10 months after fabrication. Both structures show bipolar non-volatile switching behaviour, being the ITO-based structure more stable during sweeping cycles, but presenting shorter retention of the ON state than the W-based one. Thus, further investigation of these structures taking advantage of the flexible PET/ITO substrate should be considered. The conduction mechanism in the active layer is governed by the space charge limited conduction in the HRS, trap-charge limited as $V_{SET}$ is reached and ohmic conduction processes in the LRS. We consider that this originates from an interplay of both top active electrode and the MXene flakes serving as nucleation centres for Ag^+^ ions, as well as containing oxygen vacancies terminations, creating a competing mechanism which is more pronounced in the ITO-based structure. To support this, further measurements using in situ electrical or element mapping measurements should be performed. Our analysis provides a further understating of RS devices and performance based on MXenes integration with polymers and different electrode structures, a fundamental step towards their use in this technology.

## Data Availability

The raw data supporting the conclusions of this article will be made available by the authors on request.

## Abbreviations

The following abbreviations are used in this manuscript:

2D	Two-Dimensional
FTIR	Fourier-Transform Infrared Spectroscopy
HRS	High resistance state
LRS	Low resistance state
MIM	Metal-Insulator-Metal
RS	Resistive switching
SCLC	Space Charge Limited Conduction
SEM	Scanning Electron Microscopy
XRD	X-ray Diffraction

## References

[1] Chua L. (1971). Memristor—The missing circuit element. IEEE Trans. Circuit Theory.

[2] Strukov D.B., Snider G.S., Stewart D.R., Williams R.S. (2008). The missing memristor found. Nature.

[3] Dias C., Castro D., Aroso M., Ventura J., Aguiar P. (2022). Memristor-Based Neuromodulation Device for Real-Time Monitoring and Adaptive Control of Neuronal Populations. ACS Appl. Electron. Mater..

[4] Waser R., Dittmann R., Staikov G., Szot K. (2009). Redox-Based Resistive Switching Memories—Nanoionic Mechanisms, Prospects, and Challenges. Adv. Mater..

[5] Yang J.J., Strukov D.B., Stewart D.R. (2013). Memristive devices for computing. Nat. Nanotechnol..

[6] Liu L., Xiong W., Liu Y., Chen K., Xu Z., Zhou Y., Han J., Ye C., Chen X., Song Z. (2020). Designing High-Performance Storage in HfO_2_/BiFeO_3_ Memristor for Artificial Synapse Applications. Adv. Electron. Mater..

[7] Zhang H., Jiang B., Cheng C., Huang B., Zhang H., Chen R., Xu J., Huang Y., Chen H., Pei W. (2023). A Self-Rectifying Synaptic Memristor Array with Ultrahigh Weight Potentiation Linearity for a Self-Organizing-Map Neural Network. Nano Lett..

[8] Patnaik A., Acharya A., Tiwari K., Saha P., Sahoo N., Panda D. (2024). Synaptic plasticity in zinc oxide-based flexible invisible transparent memristor by modulating oxygen concentration. J. Appl. Phys..

[9] Yang J., Zhang F., Xiao H.M., Wang Z.P., Xie P., Feng Z., Wang J., Mao J., Zhou Y., Han S.T. (2022). A Perovskite Memristor with Large Dynamic Space for Analog-Encoded Image Recognition. ACS Nano.

[10] Patel M., Kumbhar D.D., Gosai J., Sekhar M.R., Mallajosyula A.T., Solanki A. (2023). Hybrid Perovskite-Based Flexible and Stable Memristor by Complete Solution Process for Neuromorphic Computing. Adv. Electron. Mater..

[11] Cao F., Hu Z., Yan T., Hong E., Deng X., Wu L., Fang X. (2023). A Dual-Functional Perovskite-Based Photodetector and Memristor for Visual Memory. Adv. Mater..

[12] Cao G., Meng P., Chen J., Liu H., Bian R., Zhu C., Liu F., Liu Z. (2021). 2D Material Based Synaptic Devices for Neuromorphic Computing. Adv. Funct. Mater..

[13] Duan H., Cheng S., Qin L., Zhang X., Xie B., Zhang Y., Jie W. (2022). Low-Power Memristor Based on Two-Dimensional Materials. J. Phys. Chem. Lett..

[14] Nirmal K.A., Kumbhar D.D., Kesavan A.V., Dongale T.D., Kim T.G. (2024). Advancements in 2D layered material memristors: Unleashing their potential beyond memory. NPJ 2D Mater. Appl..

[15] Rehman M.M., Samad Y.A., Gul J.Z., Saqib M., Khan M., Shaukat R.A., Chang R., Shi Y., Kim W.Y. (2025). 2D materials-memristive devices nexus: From status quo to Impending applications. Prog. Mater. Sci..

[16] Teja Nibhanupudi S.S., Roy A., Veksler D., Coupin M., Matthews K.C., Disiena M., Ansh, Singh J.V., Gearba-Dolocan I.R., Warner J. (2024). Ultra-fast switching memristors based on two-dimensional materials. Nat. Commun..

[17] Ares P., Novoselov K.S. (2022). Recent advances in graphene and other 2D materials. Nano Mater. Sci..

[18] An J., Zhao X., Zhang Y., Liu M., Yuan J., Sun X., Zhang Z., Wang B., Li S., Li D. (2022). Perspectives of 2D Materials for Optoelectronic Integration. Adv. Funct. Mater..

[19] Zhang X., Chen H., Cheng S., Guo F., Jie W., Hao J. (2022). Tunable Resistive Switching in 2D MXene Ti_3_C_2_ Nanosheets for Non-Volatile Memory and Neuromorphic Computing. ACS Appl. Mater. Interfaces.

[20] Yan X., Wang K., Zhao J., Zhou Z., Wang H., Wang J., Zhang L., Li X., Xiao Z., Zhao Q. (2019). A New Memristor with 2D Ti_3_C_2_T*_x_* MXene Flakes as an Artificial Bio-Synapse. Small.

[21] Ling S., Zhang C., Ma C., Li Y., Zhang Q. (2023). Emerging MXene-Based Memristors for In-Memory, Neuromorphic Computing, and Logic Operation. Adv. Funct. Mater..

[22] Teixeira H., Dias C., Silva A.V., Ventura J. (2024). Advances on MXene-Based Memristors for Neuromorphic Computing: A Review on Synthesis, Mechanisms, and Future Directions. ACS Nano.

[23] Gogotsi Y., Anasori B. (2019). The Rise of MXenes. ACS Nano.

[24] Hart J.L., Hantanasirisakul K., Lang A.C., Anasori B., Pinto D., Pivak Y., Van Omme J.T., May S.J., Gogotsi Y., Taheri M.L. (2019). Control of MXenes’ electronic properties through termination and intercalation. Nat. Commun..

[25] Ahmad S., Ashraf I., Mansoor M.A., Rizwan S., Iqbal M. (2021). An Overview of Recent Advances in the Synthesis and Applications of the Transition Metal Carbide Nanomaterials. Nanomaterials.

[26] Gong Y., Xing X., Wang Y., Lv Z., Zhou Y., Han S.T. (2021). Emerging MXenes for Functional Memories. Small Sci..

[27] Jiang X., Kuklin A.V., Baev A., Ge Y., Ågren H., Zhang H., Prasad P.N. (2020). Two-dimensional MXenes: From morphological to optical, electric, and magnetic properties and applications. Phys. Rep..

[28] Muhammed M., Mokkath J. (2023). Surface termination dependent optical characteristics of MXene nanoflakes. Mater. Today Chem..

[29] Tang M., Li J., Wang Y., Han W., Xu S., Lu M., Zhang W., Li H. (2022). Surface Terminations of MXene: Synthesis, Characterization, and Properties. Symmetry.

[30] Lian X.J., Fu J.K., Gao Z.X., Gu S.P., Wang L. (2023). High-performance artificial neurons based on Ag/MXene/GST/Pt threshold switching memristors. Chin. Phys. B.

[31] Mao H., Gu C., Yan S., Xin Q., Cheng S., Tan P., Wang X., Xiu F., Liu X., Liu J. (2020). MXene Quantum Dot/Polymer Hybrid Structures with Tunable Electrical Conductance and Resistive Switching for Nonvolatile Memory Devices. Adv. Electron. Mater..

[32] Sang M., Liu S., Li W., Wang S., Li J., Li J., Xuan S., Gong X. (2022). Flexible polyvinylidene fluoride(PVDF)/MXene(Ti3C2Tx)/Polyimide(PI) wearable electronic for body Monitoring, thermotherapy and electromagnetic interference shielding. Compos. Part A Appl. Sci. Manuf..

[33] Qin Q., Hu Y., Guo S., Yang Y., Lei T., Cui Z., Wang H., Qin S. (2023). PVDF-based composites for electromagnetic shielding application: A review. J. Polym. Res..

[34] Wang Y., Guo T., Tian Z., Bibi K., Zhang Y.Z., Alshareef H.N. (2022). MXenes for Energy Harvesting. Adv. Mater..

[35] Kou L., Sadri R., Momodu D., Roberts E.P.L., Mohammad Haniff M.A.S., Wu C., Dee C.F., Ooi P.C. (2024). N -Doped Graphene/MXene Nanocomposite as a Temperature-Adaptive Neuromorphic Memristor. ACS Appl. Nano Mater..

[36] Wu Y., Li Y., Wang Y., Liu Q., Chen Q., Chen M. (2022). Advances and prospects of PVDF based polymer electrolytes. J. Energy Chem..

[37] Dallaev R., Pisarenko T., Sobola D., Orudzhev F., Ramazanov S., Trčka T. (2022). Brief Review of PVDF Properties and Applications Potential. Polymers.

[38] Adomaviciute-Grabusove S., Popov A., Ramanavicius S., Sablinskas V., Shevchuk K., Gogotsi O., Baginskiy I., Gogotsi Y., Ramanavicius A. (2024). Monitoring Ti_3_C_2_T_x_ MXene Degradation Pathways Using Raman Spectroscopy. ACS Nano.

[39] Cai X., Lei T., Sun D., Lin L. (2017). A critical analysis of the *α*, *β* and *γ* phases in poly(vinylidene fluoride) using FTIR. RSC Adv..

